# Mitochondria‐Targeted Temozolomide Probe for Overcoming MGMT‐Mediated Resistance in Glioblastoma

**DOI:** 10.1002/cbic.202400935

**Published:** 2025-02-26

**Authors:** Daniel Szames, Shana O. Kelley

**Affiliations:** ^1^ Department of Chemistry Department of Biomedical Engineering Department of Biochemistry and Molecular Genetics Northwestern University 2190 Campus Drive Evanston IL United States; ^2^ Department of Pharmaceutical Sciences Leslie Dan Faculty of Pharmacy University of Toronto 144 College Street Toronto ON Canada

**Keywords:** DNA Damage, Glioblastoma, Mitochondria, Peptide, Temozolomide

## Abstract

Temozolomide (Tmz) is a DNA methylating agent used for the treatment of glioblastoma multiforme (GBM). Resistance to Tmz in GBM is caused by the DNA direct repair enzyme O^6^‐methylguanine DNA methyltransferase (MGMT), which is expressed in ~50 % of GBM tumours. It has yet to be confirmed that MGMT acts within mitochondria to repair mitochondrial DNA (mtDNA), and in this report we discuss the development of a novel mitochondria‐targeted temozolomide probe (mtTmz) for evading MGMT‐mediated resistance. Through conjugation of Tmz to a mitochondria‐penetrating peptide (MPP), exclusive mitochondrial localization was achieved, and the probe retained alkylation activity demonstrated by chemical and DNA‐based assays. Absence of nuclear DNA damage was assessed by detecting γH2AX foci. mtTmz demonstrated efficient cell killing capabilities independent of MGMT status in GBM cells as determined by cell viability assays. It was determined using a Proteinase K digestion assay that MGMT does not translocate to mitochondria in response to mtTmz treatment, and RT‐qPCR analysis demonstrated that mtTmz does not induce MGMT gene expression compared to Tmz. The results reported highlight both the potential of mitochondrial targeting of Tmz and mitochondria as a therapeutic target in MGMT‐expressing GBM.

## Graphical abstract

Mitochondria‐targeted temozolomide (mtTmz) using peptide‐based delivery vector has been shown to overcome direct DNA repair and resistance by O^6^‐methylgunaine DNA methyltransferase (MGMT) in glioblastoma (GBM) cells. This has been demonstrated through viability assays using MGMT‐positive and negative cell lines, and the effect of mtTmz on MGMT subcellular localization and expression was analysed using Proteinase K digestion assays and RT‐qPCR, respectively.

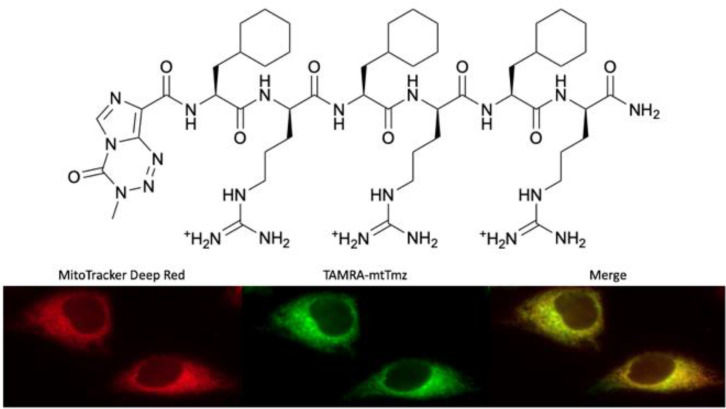

## Introduction

Glioblastoma multiforme (GBM) is the most common and lethal brain tumour affecting the central nervous system in adults. Prognosis is typically poor with an associated overall survival of less than one year.^[1]^ The current standard‐of‐care for patients newly diagnosed with GBM is surgical resection of the bulk tumour, followed by radiotherapy and concomitant chemotherapy.^[2]^ The chemotherapeutic agent that has been the most successful in GBM treatment is the DNA methylating agent temozolomide (Tmz). Tmz is a small molecule imidazotetrazine prodrug, and under physiological conditions undergoes hydrolysis to yield the active metabolite 5‐(3‐methyltriazen‐1‐yl)imidazole‐4‐carboxamide (MTIC), which is further metabolized to 5‐aminoimidazole‐4‐carboxamide (AIC) and the electrophilic methyldiazonium ion. Tmz induces DNA‐specific methylated adducts, namely N^3^‐methyladenine (9 %), N^7^‐methylguanine (70 %), and O^6^‐methylguanine (6 %).^[3]^ Despite being the least abundant DNA adduct formed, O^6^‐methylguanine is mainly responsible for the cytotoxic effect of Tmz. Whereas N^3^‐methyladenine and N^7^‐methylguanine are readily repaired by base excision repair (BER), O^6^‐methylguanine forms DNA mismatches with thymine during replication, triggering a futile cycle of the mismatch repair (MMR) pathway, collapse of the replication fork, and DNA double‐strand breaks.^[4]^ Therefore, GBM cells proficient in MMR respond positively to Tmz therapy, whereas cells lacking MMR machinery tolerate the damage and are resistant to treatment. Additionally, it has been shown that the DNA repair protein O^6^‐methylguanine‐DNA methyltransferase (MGMT), which transfers the methyl group from cytotoxic O^6^‐methylguanine adducts to itself, confers resistance to Tmz treatment in GBM. Following this repair process, MGMT is irreversibly inactivated and subsequently degraded by the proteasome.^[5]^ It has been reported that in about 50 % of GBM tumours there is active MGMT expression, corresponding to hypomethylation of the MGMT promoter, whereas in the other 50 %, MGMT expression is silenced due to promoter hypermethylation. Patients with hypomethylated MGMT promoters and active MGMT expression derive little benefit from Tmz treatment and are often met with a poor prognosis.^[6]^

Recent efforts have focused on designing Tmz derivatives to bypass either MGMT or MMR‐mediated resistance in GBM cells.[^7^, ^8^] These approaches use mechanism‐based drug design to synthesize analogues of Tmz that result in inducing unique lesions on DNA that are either not repaired by MGMT or not dependent on MMR status. These studies suggest that therapeutic agents could be modified to cause damage that is irreparable by DNA repair factors, ultimately resulting in cell death.

Mitochondria are another organellar compartment of the cell that contain their own DNA molecule (mtDNA). mtDNA is a small circular DNA molecule of ~16.6 kb that strictly encodes essential protein subunits of the electron transport chain (ETC) necessary for generating energy via oxidative phosphorylation (OXPHOS), and the RNA molecules associated with synthesizing such proteins, and is located within the tightly protected mitochondrial matrix.[^9^, ^10^] As mtDNA only encodes for protein subunits of the ETC, all other mitochondrial proteins (including those involved in the repair and replication of mtDNA) are nuclear encoded, synthesized on cytosolic ribosomes, and subsequently translocated to mitochondria. Furthermore, it has been shown that mitochondria do not possess the same DNA repair and replication machinery that exist in the nucleus, leaving mtDNA more vulnerable to insults.[^9^, ^10^] Due to the proximity of mtDNA to the site of ROS production, the most characterized DNA repair pathway observed in mitochondria is BER carried out by the nuclear encoded protein 8‐oxoguanine glycosylase (OGG1).^[11]^ There has been evidence of other pathways acting in mitochondria, such as double‐strand break (DSB) repair,^[12]^ single‐strand break (SSB) repair,^[13]^ and MMR,^[14]^ all via nuclear encoded proteins. It has been shown that MGMT does not endogenously localize to mitochondria, and its role in repairing mtDNA has yet to be confirmed.^[15]^ The relative vulnerability of mtDNA to exogenously induced damage opens the possibility of redirecting DNA damaging agents to mitochondria to overcome repair and resistance mechanisms. However, accessing the mitochondrial matrix where mtDNA is housed has proven to be a challenging obstacle to many small molecule DNA damaging molecules due to the impenetrable double membrane of the organelle.

To overcome this barrier, cationic and amphiphilic vectors have been successfully employed to achieve mitochondrial localization.[^16^, ^17^] Efforts by our group have resulted in the development of a mitochondria‐penetrating peptide (MPP) that demonstrates exclusive localization within the mitochondrial matrix.^[18]^ A suite of MPP‐drug conjugates has been used to deliver a variety of small molecule DNA‐damaging agents to the mitochondrial matrix.[^19^, ^20^, ^21^, ^22^] Using this platform, our group has shown that mitochondrial delivery of anticancer agents can overcome resistance mechanisms such as drug efflux pumps and regulation of apoptosis. Herein, we discuss the development of a novel mitochondria‐targeted temozolomide probe (mtTmz) based on a MPP, evaluate its alkylation profile, determine the effect of MGMT expression on its activity in GBM cells, and confirm lack of MGMT recruitment to mitochondria in Tmz‐treated GBM cells. This report highlights the potential of mitochondria and mtDNA as a potential therapeutic target for Tmz in MGMT‐expressing GBM.

## Results and Discussion

### Design and Synthesis of mtTmz

**Figure 1:**
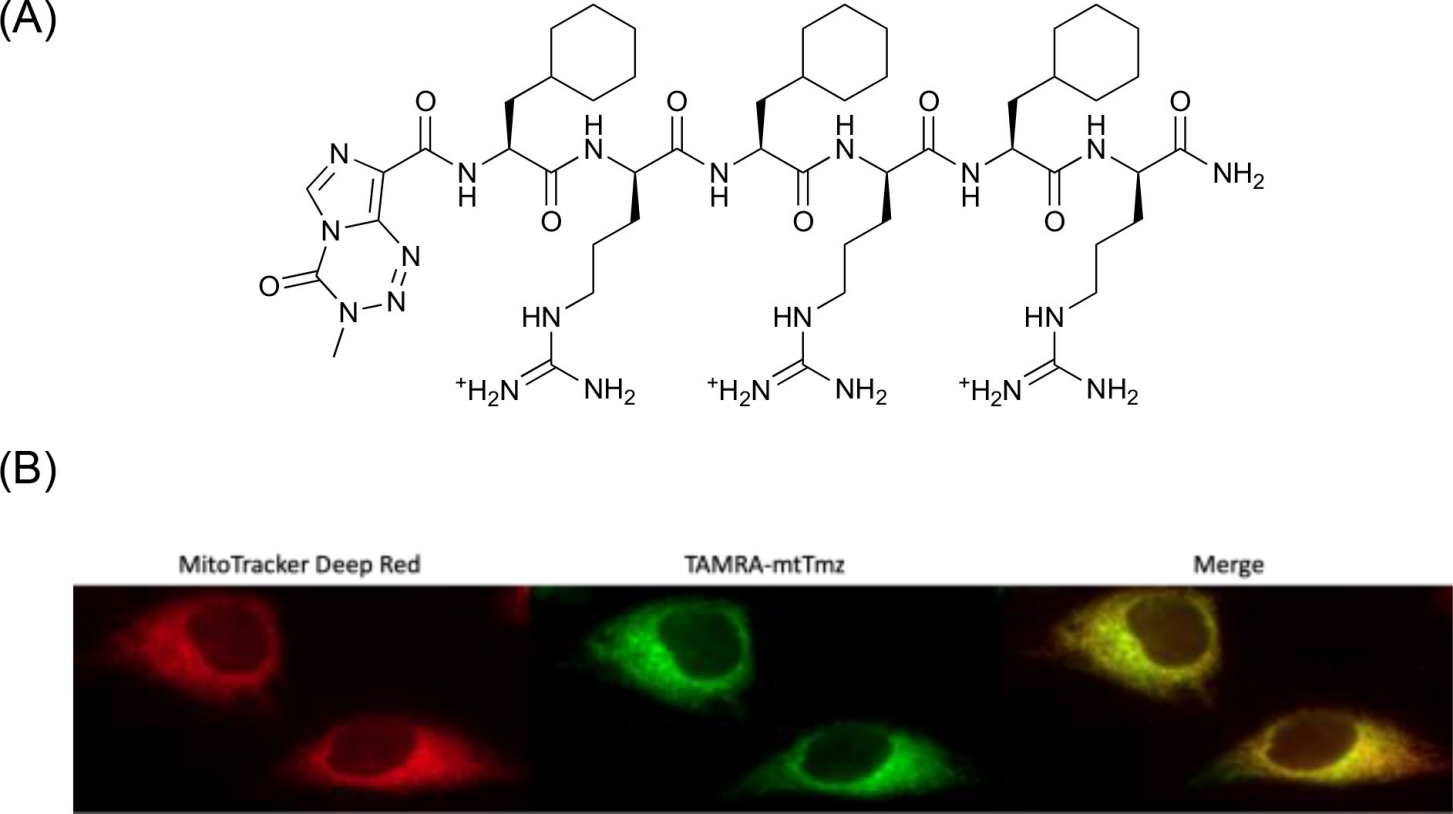
(A) Molecular structure of mtTmz. Temozolomide acid is conjugated to the mitochondria‐penetrating peptide (MPP). (B) Live cell fluorescence microscopy images showing co‐localization of mtTmz (green) and mitochondria (red) in HeLa cells. Images taken after 1 hour treatment with 10 uM TAMRA‐mtTmz. Mitochondria are stained using 150 nM MitoTracker Deep Red.

Mitochondrial localization can be achieved with a six‐amino acid peptide with repeating alternating units of cyclohexylalanine (F_x_) and d‐arginine (r).[^18^, ^23^] (F_x_r)_3_ is synthesized via solid‐phase peptide synthesis (SPPS); Tmz is conjugated to the N‐terminal primary amine using the carboxylic acid derivative (Tmz acid), and subsequently cleaved from the solid‐phase resin (Scheme S1). Use of the Tmz acid derivative does not affect activity of the drug as the pharmacological activity of Tmz is linked to the tetrazine moiety of the molecule. Mass spectrometry and HPLC characterization of mtTmz are shown in Figures S1 and S2, respectively. The molecular structure of mtTmz is shown in Figure 1A. A fluorescently labelled mtTmz was synthesized for use in fluorescence microscopy imaging to confirm subcellular localization by conjugating 5,6‐carboxytetramethylrhodamine (TAMRA) to a lysine linker on mtTmz (Scheme S2). Mass spectrometry and HPLC characterization of TAMRA‐mtTmz are shown in Figures S3 and S4, respectively. mtTmz demonstrates exclusive mitochondrial localization in HeLa cells, as can be seen in Figure 1B, showing co‐localization of mtTmz with the MitoTracker Deep Red stain.

### DNA Alkylation & Damage Properties of mtTmz

**Figure 2:**
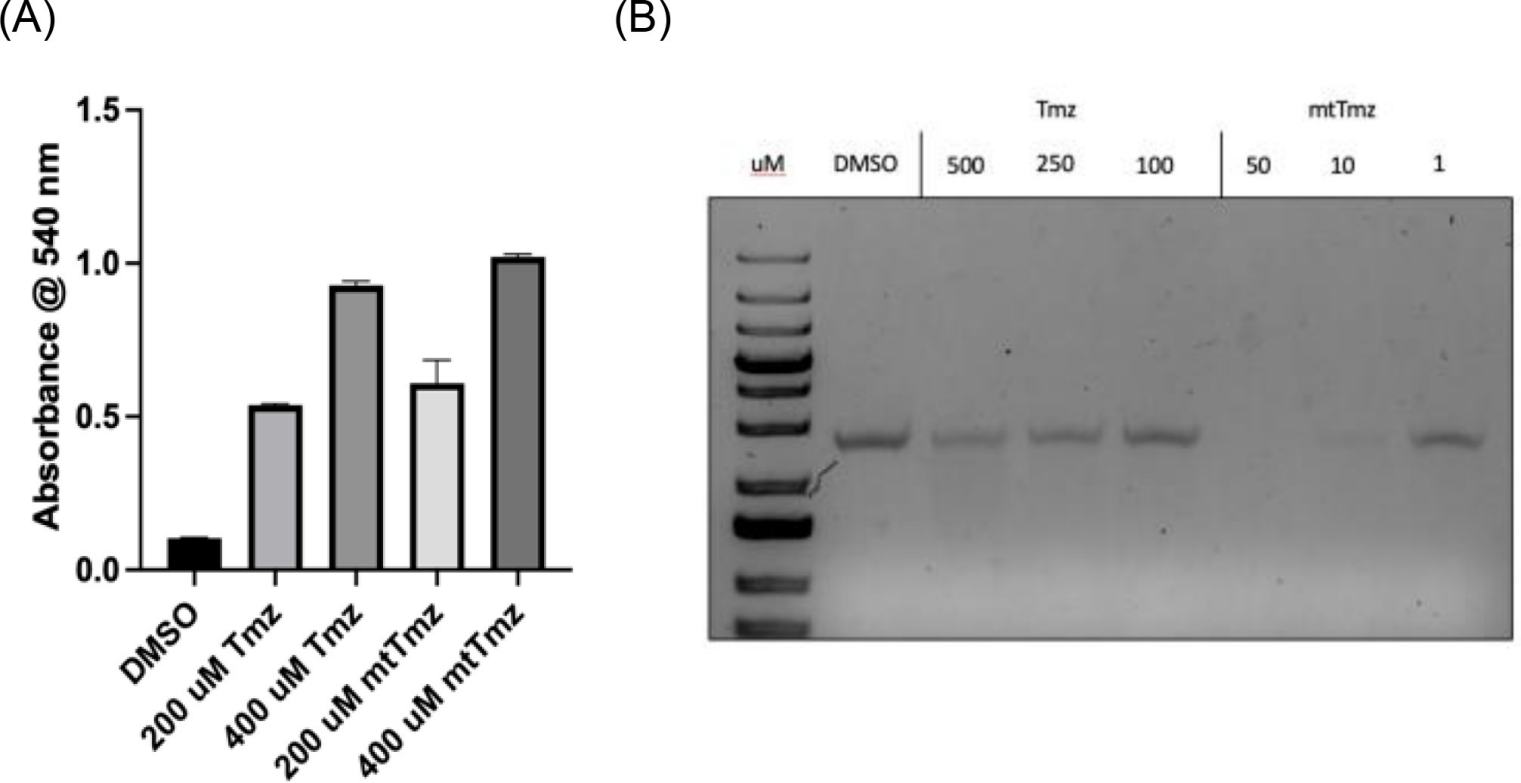
Alkylation and DNA damage properties of mtTmz. (A) Chemical alkylation assay using 4‐NBP demonstrating alkyaltion activity of both Tmz and mtTmz after 24 hours. (B) DNA alkylation assay comparing Tmz and mtTmz using linearized pBR322 plasmid DNA. Conditions: linearized pBR322 DNA (50 ng), 37 °C, water, overnight. DNA visualized on a 1 % agarose gel with ethidium bromide staining.

To assess the alkylation activity of mtTmz, a chemical alkylation assay using 4‐(4‐nitrobenyzl)pyridine (4‐NBP) was used (Figure 2A). In this assay, the indicated compounds are incubated with 4‐NBP under physiological conditions (pH 7.4; 37 °C), and upon alkylation, 4‐NBP undergoes a colour change that can be measured at 540 nm.^[24]^ Compared to Tmz, mtTmz demonstrated a similar alkylation profile, with a direct correlation between concentration and rate of alkylation after 24 hours of incubation. Next, to determine the effect of mtTmz on DNA alkylation, the biochemical assay employing linearized pBR322 plasmid DNA was used (Figure 2B).^[25]^ After linearization with EcoRI‐HF and column purification, plasmid DNA was incubated with the indicated compounds overnight at 37 °C and subsequently denatured and analysed on a 1 % agarose gel (Figure 2B). DNA streaking on the gel corresponds to shorter fragments being formed after alkaline denaturation of alkylated DNA, and this can be seen with both compound treatments, markedly more with mtTmz.

**Figure 3:**
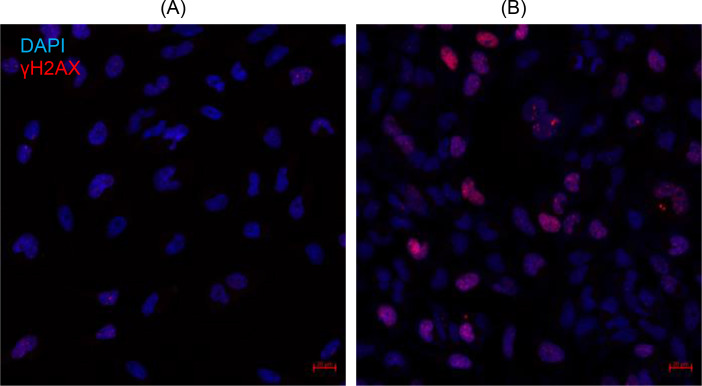
Confocal microscopy images of γ‐H2AX foci (red) in HeLa treated with 100 uM (A) mtTmz and (B) Tmz, Nuclei are stained with DAPI (blue). Scale bar is 20 um.

To confirm a lack of nuclear DNA damage caused by mtTmz, the presence of γH2AX foci was measured using confocal microscopy (Figure 3). In the microscopy images shown, there is a significant increase in foci in HeLa cells treated with 100 uM Tmz after 24 hours compared to cells treated with mtTmz under the same conditions. Taken together, these data demonstrate the DNA alkylation activity of mtTmz, while leaving nuclear DNA intact.

### Effect of MGMT Status on mtTmz Activity in GBM Cells

**Figure 4:**
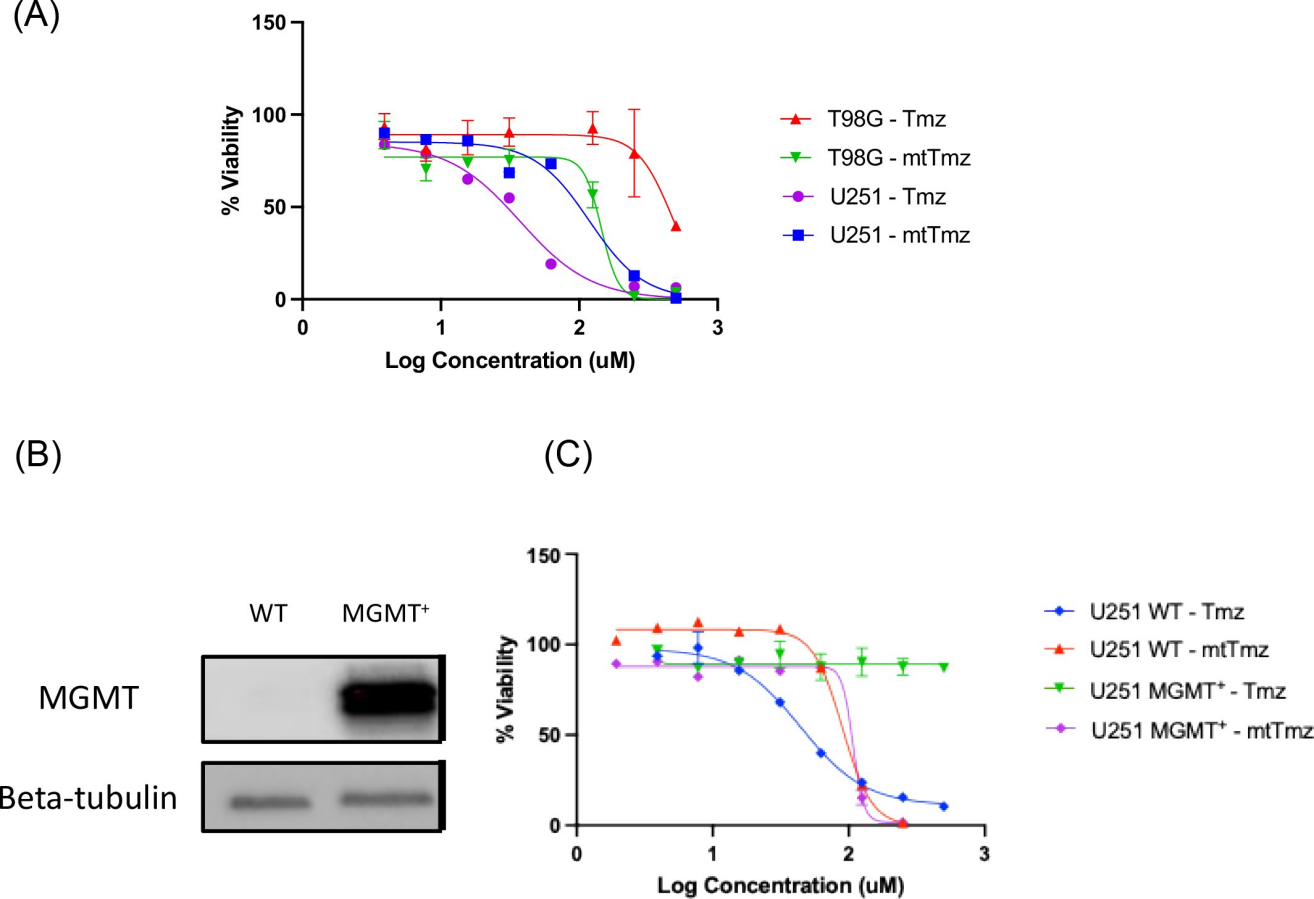
Effect of MGMT protein expression on mtTmz activity. (A) Cell viability of T98G (MGMT^+^) and U251 (MGMT^−^) cells treated with both Tmz and mtTmz. Viability measured after 7 days with CCK8 viability dye. (B) MGMT protein expression in U251 cells transfected with MGMT expression vector compared to wild type (WT) cells. (C) Cell viability of U251 MGMT^+^ cells treated with both Tmz and mtTmz. Viability measured after 7 days with CCK8 viability dye.

To determine whether MGMT expression in GBM cells affects the activity of mtTmz, cell viability experiments were conducted comparing the viability of both MGMT‐negative (MGMT^−^), and MGMT‐positive (MGMT^+^) GBM cells treated with both Tmz and mtTmz. MGMT status has an observable effect on the activity of Tmz, with T98G cells (MGMT^+^) being resistant and U251 cells (MGMT^−^) being sensitive to the drug after 7 days of treatment (Figure 4A). However, MGMT status does not seem to potentiate the activity of mtTmz, as the compound's potency remains relatively unchanged in both cell lines. The potency of mtTmz and the peptide‐vector (Fxr)_3_ was also compared in HeLa cells, showing the increased activity of the peptide‐drug conjugate compared to the delivery vector after 48‐hours of treatment (Figure S6).

To further investigate the relationship between MGMT expression and cell killing activity of mtTmz, a U251 MGMT‐expressing cell line was generated (U251 MGMT^+^) (Figure 4B). Similarly, expressing MGMT in this cell line causes strong resistance to Tmz, whereas the potency of mtTmz remains unchanged in both cell lines (Figure 4C). To determine if the observed effect in the U251 MGMT^+^ cells is a result of MGMT expression, the selective MGMT inhibitor O^6^‐benzylguanine (O^6^BG) was used. 3‐ and 5‐day cell viability was measured in cells treated with 200 and 400 uM Tmz both with and without 3‐hour pretreatment with 100 uM O^6^BG. (Figure S7). Cells with O^6^BG pretreatment became sensitized to Tmz treatment compared to cells with no inhibitor pretreatment, indicating rescue of this cell line to Tmz treatment is due to MGMT activity. These results demonstrate that MGMT expression has no observable effect on the cell killing capabilities of mtTmz in GBM cells, suggesting lack of MGMT recruitment to mitochondria in response to mtTmz treatment.

### Evasion of MGMT Recruitment to mtTmz‐induced mtDNA Damage

**Figure 5:**
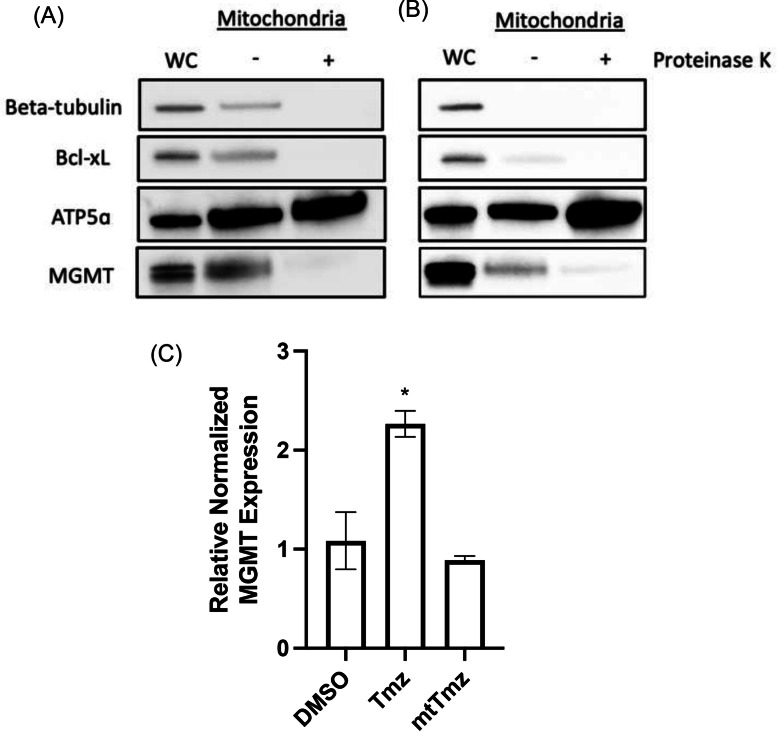
Effect of mtTmz on mitochondrial MGMT expression and gene expression. U251 MGMT^+^ cells were either (A) untreated or (B) treated with 50 uM mtTmz for 24 hours, after which mitochondria were isolated and either suspended in buffer alone or buffer containing 100 ug/mL Proteinase K and separated based on size using SDS‐PAGE. Bcl‐xL is a mitochondrial outer membrane protein. ATP5α is a mitochondrial matrix protein. Beta‐tubulin acts as whole cell (WC) lysate control. (C) RT‐qPCR analysis of MGMT gene expression in T98G cells after 24 hours treatment with DMSO, 100 uM Tmz, and 50 uM mtTmz.

Based on the results above demonstrating no observable effect of MGMT on the cell kill capabilities of mtTmz, the response of MGMT to mtTmz treatment was investigated. To determine whether MGMT is recruited to mitochondria after mtTmz treatment, a Proteinase K digestion assay was conducted (Figure 5A & B). Proteinase K digests proteins on the outer membrane of isolated mitochondria, whereas proteins located inside the mitochondrial matrix are protected. Mitochondria were isolated from U251 MGMT^+^ cells that were either untreated or treated with 50 uM mtTmz overnight, followed by digestion with 100 ug/mL Proteinase K. Under mtTmz treated conditions, there is negligible translocation of MGMT to mitochondria, as demonstrate by Western blot analysis. Additionally, mtTmz did not show to cause any mitochondrial translocation in HeLa cells, which are endogenous MGMT expressing cells (Figure S8).

mtTmz treatment on MGMT expression was also investigated at the gene level using RT‐qPCR (Figure 5C). Compared to Tmz, mtTmz does not show to increase MGMT transcript expression in T98G GBM cells, suggesting this type of damage on mtDNA does not induce expression of MGMT.

## Conclusions

The lack of DNA repair processes in mitochondria compared to the nucleus renders mtDNA more vulnerable to DNA damaging molecules, and therefore poses as a potential alternative target for these drugs. Achieving successful delivery of DNA damaging drugs to the mitochondria has proven challenging due to the highly selective double‐membrane surrounding the matrix where the genome is housed. This work highlights the development of a novel mitochondria‐targeted temozolomide probe using a peptide‐based delivery vector for inducing DNA‐specific methylation damage in mitochondria and bypassing DNA damage repair by MGMT. It was demonstrated that the effect of mtTmz on the viability of GBM cells remains the same regardless of cellular MGMT status. Additionally, it was shown that mtTmz treatment does not recruit MGMT translocation to mitochondria using Proteinase K digestion assays or induce MGMT expression as shown by RT‐qPCR. Taken together these results highlight the potential of targeting mitochondria and mtDNA as a therapeutic approach for MGMT‐expressing GBM. The development of mtTmz also allows for further studies involving MGMT in mitochondria, as well as other DNA repair pathways, for example investigating mismatch repair in the organelle.

## Experimental Section

**Cell Culture**: All cell lines used were cultured in Eagle's Minimum Essential Medium (EMEM, Wisent) supplemented with 10 % fetal bovine serum (v/v) (FBS, Invitrogen), 1 % penicillin‐streptomycin (v/v) (10,000 U/mL, Gibco), and incubated at 37 °C with 5 % CO_2_. U251 GBM cells were generously donated by Dr. Raymond Reilly's research group at the Leslie Dan Faculty of Pharmacy, University of Toronto.

**Synthesis and Characterization of mtTmz**: Peptide ((Fxr)_3_) was synthesized using an automated Prelude peptide synthesizer (Protein Technologies) as described previously.^[26]^ To 50 umol (Fxr)_3_ on Rink amide resin (Novabiochem) in a Poly‐Prep chromatography column (Bio‐Rad), a 1 mL conjugation reaction solution of Tmz acid (2 eq., Toronto Research Chemicals), *O*‐(benzotriazol‐1‐yl)‐*N,N,N′,N′*‐tetramethyluronium hexafluorophosphate (HBTU, 2 eq., Sigma Aldrich), and *N,N*‐diisopropylethylamine (DIPEA, 6 eq., Sigma Aldrich) was added and the reaction was placed on a shaker overnight. The resin was then washed with *N,N*‐dimethylformamide (DMF, Fisher Chemical), methanol (Caledon), and dichloromethane (DCM, Fisher Chemical) and dried over vacuum. To cleave mtTmz from resin, a mixture of trifluoroacetic acid (TFA, Sigma Aldrich):triisoproylsilane (TIPS, Sigma Aldrich):deionized water (95 : 2.5 : 2.5 v/v) was added to dried resin and placed on shaker for 2 hours. Cleaved peptide solution was precipitated in 40 mL of cold diethyl ether (Caldeon) and placed at −20 °C for at least 1 hour before being retrieved by centrifugation at 3000 g for 10 minutes. Crude mtTmz was dissolved in 3 mL of 30 % acetonitrile in water, filtered using a 0.22 um syringe filter, and purified using preparative RP‐HPLC on a C_18_ column. Mobile phase gradient was 30–50 % acetonitrile (0.1 % TFA) in water (0.1 % TFA). Purified mtTmz was dried using a rotary evaporator and lyophilized. mtTmz was dissolved in dimethyl sulfoxide (DMSO, Sigma Aldrich), and was characterized using electrospray ionization mass spectrometry (ESI/MS) and quantified using a bicinchoninic acid (BCA) assay (Thermo Scientific). ESI/MS (positive ion mode): *m/z*=1121.70.

**Synthesis and Characterization of TAMRA labelled mtTmz (TAMRA‐mtTmz)**: (Fxr)_3_ with lysine linker (K) protected with 4‐methyltrityl (Mtt) was used for synthesis of TAMRA‐conjugated peptide ((Fxr)_3_K‐Mtt). First, Tmz conjugation at the N‐terminus was performed as described above to (Fxr)_3_K‐Mtt (25 umol) on resin. After washing resin with DMF, 3 % TFA in DCM (1 mL) was added to deprotect the Mtt and placed on shaker for 30 minutes. Resin was then washed with DMF. To the resin, a solution of 5(6)‐carboxytetramethylrhoadmine (TAMRA, 2 eq., Anaspec), HBTU (2 eq.), and DIPEA (6 eq.) in DMF (1 mL) was added and placed on shaker overnight. Peptide was cleaved and purified as described above. TAMRA‐mtTmz was quantified by spectrophotometry (Molecular Devices) using a molar extinction coefficient of 90,000 M^−1^cm^−1^ at 553 nm in water. ESI/MS (positive ion mode): *m/z*=1662. 95.

**Live Cell Imaging**: HeLa cells were seeded in a 8‐well μSlide (ibidi) at 10,000 cells/well and allowed to adhere overnight. Cells were then treated with 10 uM TAMRA‐mtTmz in serum‐free Opti‐MEM media (Gibco) for 1 hour at 37 °C with 5 % CO_2_. The media was then replaced with Opti‐MEM containing 150 nM MitoTracker Deep Red (Invitrogen) for 20 minutes under the same conditions before two washes with PBS and imaged using an inverted Zeiss confocal microscope.

**Colourimetric Alkylation Assay**: A modified method of the 4‐(4‐nitrobenzyl)pyridine assay described previously was performed to measure alkylation activity of the discussed compounds.^[24]^ Briefly, each compound (50 uL) was added in the appropriate concentration to PBS (250 uL), followed by addition of 200 uL 5 % (w/w) 4‐NBP (Fisher Scientific) dissolved in acetone. Tubes were mixed thoroughly and placed at 37 °C overnight. Tubes were then chilled on ice before addition of 5 : 2 ethyl acetate:acetone (200 uL). To one tube at a time, 5 N NaOH was added (25 uL), vortexed for 10 seconds, and after separation of the phases, 100 uL of the top (organic) layer was added to a cuvette and absorbance was measured at 540 nm using a spectrophotometer (Molecular Devices). 5 : 2 ethyl acetate:acetone was used as a blank.

**Agarose Gel DNA Alkylation Assay**: Method was adapted from Healy et al.^[25]^ Briefly, pBR322 plasmid DNA (New England Biolabs) was linearized with EcoRI‐HF in cutsmart buffer (New England Biolabs) according to manufacturer's instructions. Cut plasmid DNA was purified using PureLink PCR purification kit (Thermo Fisher Scientific) and eluted in water. Purified plasmid DNA was quantified using a NanoDrop 1000 spectrophotometer (Thermo Fisher Scientific). Plasmid DNA (50 ng) and appropriate amount of compound (5 % DMSO, v/v) were added to reaction tubes, and topped up to 10 uL with sterile water. Reaction tubes were incubated at 37 °C overnight. Upon completion, peptide‐treated DNA was subject to phenol‐chloroform extraction to remove peptide bound from DNA, as it was seen that DNA was stuck at top of gel with peptide treatment. This was performed using UltraPure Phenol:Chloroform:Isoamyl alcohol (25 : 24 : 1, v/v) (Thermo Fisher Scientific), following the manufacturer's instruction. Following DNA extraction from peptide, all DNA samples were denatured using 30 uL of DNA denaturation buffer (60 mg/mL sucrose, 10 mg/mL NaOH, and 0.4 mg/mL bromophenol blue in water). Samples were vortexed and denatured for 15 minutes at 4 °C before being loaded onto a 1 % agarose gel in TAE buffer (both containing 0.5 ug/mL ethidium bromide) and ran at 100 V for 1 hour. Gels were imaged using a BioRad Chemidoc imager.

**γH2AX Foci Assay**: HeLa cells were seeded in a 8‐well μSlide (ibidi) at 8,000 cells/well and allowed to adhere overnight. Cells were then treated with the indicated compounds in 5 % FBS containing media for 24 hours. After treating, cells were washed twice with PBS, fixed with 4 % paraformaldehyde in PBS for 15 minutes at room temperature. Cells were permeabilized with 0.4 % Triton X‐100 in PBS for 15 minutes at room temperature. Cells were blocked for 1 hour at room temperature in blocking buffer (5 % FBS and 0.1 % Trion X‐100 in PBS), followed overnight incubation at 4 °C in primary anti‐γH2AX antibody (Cell Signalling Technology, 9718S) diluted 1 : 400 in blocking buffer. Cells were washed twice with PBS and incubated for 1 hour at room temperature in Alexa Fluor 647 anti‐rabbit secondary antibody (Thermo Fisher Scientific, A‐21245) 1 : 1000 in PBS. Cells were washed twice with PBS and mounted in 0.1 ug/mL DAPI in PBS and imaged using an inverted Zeiss confocal microscope at 20x magnification.

**Cell Viability Assay**: For cell viability measurements, cells were seeded in 96‐well plates at 5,000‐10,000 cells/well and adhered overnight. Cells were treated with compounds the following day in 5 % FBS containing media and treated for the indicated time points. After treatment, drug‐containing media was removed, and Cell Counting Kit‐8 (CCK‐8, Dojindo Laboratories) was added diluted 1 : 10 in Opti‐MEM media and placed at 37 °C for 1 hour. Absorbance was measured using plate reader (Molecular Devices) at 450 nm.

**Stable Cell Line Generation**: MGMT human tagged ORF clone was purchased from Origene Technologies Inc., Rockville, MD (RC229131). General transfection protocol using polyethylenimine (PEI) and Opti‐MEM media was followed from addgene. Protocol was adapted for transfection of cells in a 6‐well plate. U251 GBM cells (MGMT negative) were used as the host. Transfected cells were selected for using 300 ug/mL Geneticin G418 sulphate (Thermo Fisher Scientific, 10131035) in complete EMEM media. MGMT expression in U251 transfected cells was confirmed by Western Blotting as described below.

**Western Blotting**: Cells were harvested by trypsinization, pelleted, and washed with PBS. Cells were then lysed in RIPA buffer containing 1X protease/phosphatase inhibitor cocktail (Cell Signalling Technology) and placed either on a rotator at 4 °C for 30 minutes or at −20 °C overnight. Following lysis, cells were centrifuged at 16,000 g for 15 minutes at 4 °C, and soluble protein in supernatant was collected. BCA assay was used to quantify sample protein concentrations, and to 20 ug of protein, Laemmli sample buffer containing β‐mercaptoethanol was added and boiled at 95 °C for 5 minutes. Protein samples were loaded onto a 4–15 % Tris‐glycine gradient gel and ran at 150 V in Tris/Glycine/SDS buffer (Bio‐Rad). Proteins were transferred onto a PVDF membrane in Tris/Glycine + 20 % methanol buffer for 1 hour at 100 V. Membranes were blocked in 5 % skim milk TBS−T for 1 hour at room temperature. Membranes were then incubated in blocking buffer containing primary antibodies overnight at 4 °C. Primary antibodies used and dilutions were as follows: anti‐MGMT 1 : 500 (EMD Millipore, MAB16200); anti‐Beta‐tubulin 1 : 1000 (Cell Signalling Technology, 2128S); anti‐Bcl‐xL 1 : 500 (Abcam, ab32370); anti‐ATP5ɑ 1 : 1000 (Abcam, ab14748). Membranes were washed three times in TBS−T and incubated for 1 hour at room temperature in anti‐rabbit or anti‐mouse HRP‐linked secondary antibodies (Cell Signalling Technology, 7074/6S) 1 : 2500 in TBS−T. Membranes were again washed three times in TBS‐TT and developed using SuperSignal West Pico PLUS Chemiluminescent substrate (Thermo Fisher Scientific) and imaged using a BioRad Chemidoc imager.

**Proteinase K Digestion Assay**: A modified Proteinase K digestion method as described previously was performed.^[27]^ Briefly, U251 MGMT^+^ cells were seeded in 15 cm culture dishes and adhered overnight. Cells were then treated overnight with either 50 uM mtTmz or the same volume of DMSO in 5 % FBS media. Cells were also treated in 10 cm culture dishes for whole cell lysate collection, and those samples were prepared as described above. Cells for mitochondrial isolation were harvested by trypsinization, washed with PBS, and mitochondria were isolated using the Mitochondria Isolation Kit for Cultured Cells (Thermo Fisher Scientific) following manufacturer's protocol for physical homogenization. Mitochondrial protein concentration was quantified using BCA assay. 40 ug of mitochondrial protein for each sample was added to an equal volume of 250 mM sucrose in TE buffer either with or without 200 ug/mL Proteinase K and placed on ice for 20 minutes. Two volumes of ice cold 20 % trichloroacetic acid (TCA) was added and kept on ice for another 30 minutes to precipitate proteins. Proteins were centrifuged at 12,000 g for 5 minutes, followed by a wash with 200 uL ice cold acetone. Mitochondrial protein pellets were placed at 95 °C for 5 minutes to evaporate acetone. 40 uL Laemmli sample buffer was added to protein samples, and Western blotting procedure as described above was performed.

**RT‐qPCR**: T98G GBM cells were seeded 100,000 cells/well in a 6‐well plate and adhered overnight. Cells were treated with indicated compounds for 24 hours in 5 % FBS media, and then harvested by trypsinization. Total RNA was extracted from cells using the RNeasy Mini Kit (Qiagen, 74104) according to manufacturer's protocol and quantified via nanodrop. 1 ug of purified RNA was used to synthesize cDNA using the SuperScript VILO cDNA Synthesis Kit (Thermo Fisher Scientific, 11754050) following manufacturer's protocol. qPCR was performed using SYBR Green chemistry (Thermo Fisher Scientific, 4309155) on a BioRad CFX384 Real‐Time PCR System and normalized to GAPDH. Primers were purchased from Integrated DNA Technologies and sequences were as follows: MGMT – F: TTTTCCAGCAAGAGTCGTTCAC, R: GGGACAGGATTGCCTCTCAT; GAPDH – F: GTCTCCTCTGACTTCAACAGCG, R: ACCACCCTGTTGCTGTAGCCAA.

## Conflict of Interests

The authors declare no conflict of interest.

1

## Supporting information

As a service to our authors and readers, this journal provides supporting information supplied by the authors. Such materials are peer reviewed and may be re‐organized for online delivery, but are not copy‐edited or typeset. Technical support issues arising from supporting information (other than missing files) should be addressed to the authors.

Supporting Information

## Data Availability

The data that support the findings of this study are available from the corresponding author upon reasonable request.
