# Supplementary material for: Mitochondria‐Targeted Temozolomide Probe for Overcoming MGMT‐Mediated Resistance in Glioblastoma
Source: Chembiochem. 2025 Feb 26;26(6):e202400935. doi: 10.1002/cbic.202400935 (PMC11907389; doi:10.1002/cbic.202400935)
Supplement: Supplementary file 1 — Supporting Information [file CBIC-26-e202400935-s001.pdf]

ChemBioChem

Supporting Information

Mitochondria-Targeted Temozolomide Probe for Overcoming MGMT-Mediated Resistance in Glioblastoma

Daniel Szames and Shana O. Kelley*

Supporting Information

Mitochondria-Targeted Temozolomide Probe for Overcoming MGMT-Mediated Resistance in Glioblastoma

Daniel Szames[b] and Shana O. Kelley*[a,b]

[a] Dr. Shana O. Kelley
Department of Chemistry, Department of Biomedical Engineering, Department of Biochemistry and Molecular Genetics
Northwestern University
2190 Campus Drive, Evanston, IL United States
Email: shana.kelley@northwestern.edu

[b] Daniel Szames, Dr. Shana O. Kelley
Department of Pharmaceutical Sciences
Leslie Dan Faculty of Pharmacy
University of Toronto
144 College Street, Toronto, ON Canada

Table of Contents

Supporting Information Schemes

Scheme S1. mtTmz synthesis……………………………………………………………3

Scheme S2. TAMRA-mtTmz synthesis……………………………………………………5

Supporting Information Figures

Figure S1. mtTmz mass spectrum…………………………………………………………4

Figure S2. mtTmz HPLC chromatogram…………………………………………………..4

Figure S3. TAMRA-mtTmz mass spectrum ………………………………………………6

Figure S4. TAMRA-mtTmz HPLC chromatogram…………………………………………6

Figure S5. DNA-based alkylation assay with $(Fxr)_3$ ……………………………………...7

Figure S6. Cell viaiblity experiment with $(Fxr)_3$……………………………………………8

Figure S7. Cell viability of U251 $MGMT^+$ with O^6BG ……………………………………..9

Figure S8. Proteinase K digestion assay in HeLa cells……………………………………10

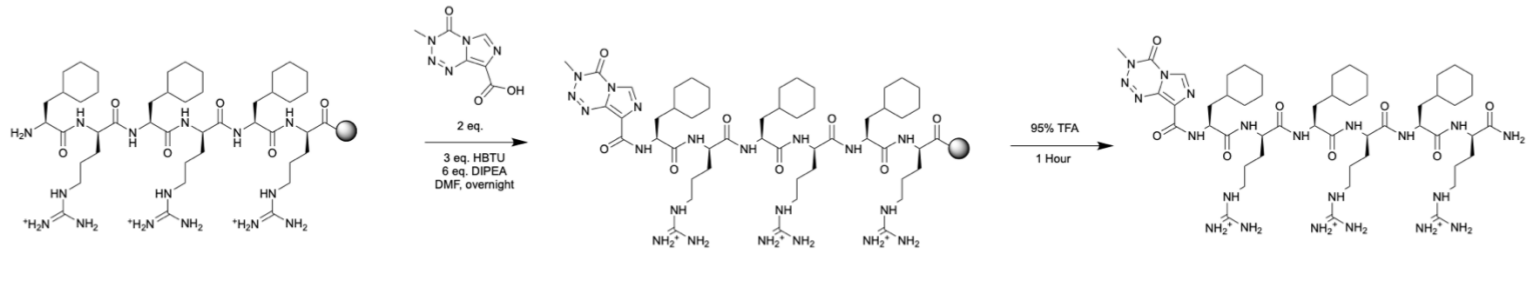

Scheme S1. mtTmz synthesis. The sphere represents the solid bead resin containing the peptide until it is cleaved by concentrated TFA after Tmz conjugation.

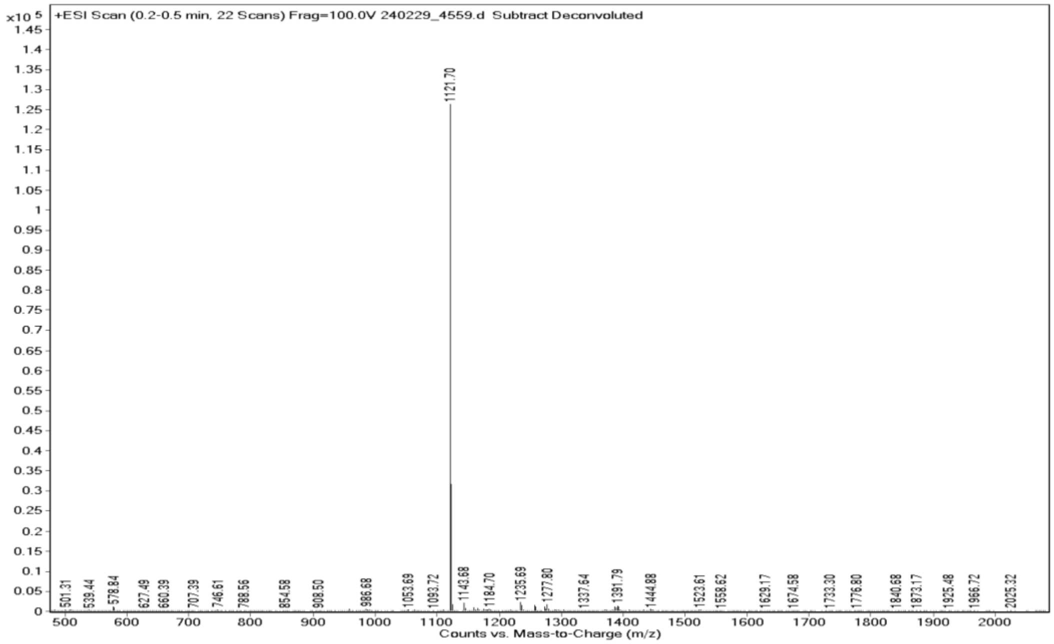

Figure S1. Mass spectrum for mtTmz.

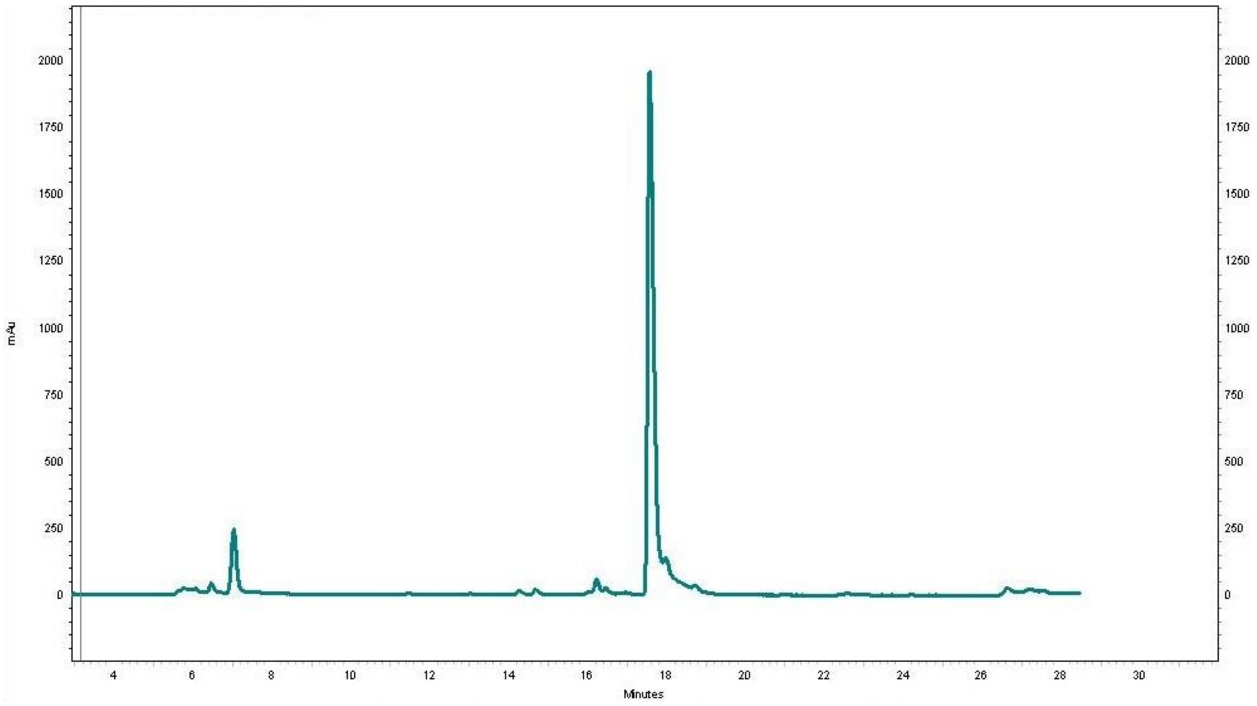

Figure S2. HPLC chromatorgram of pure mtTmz.

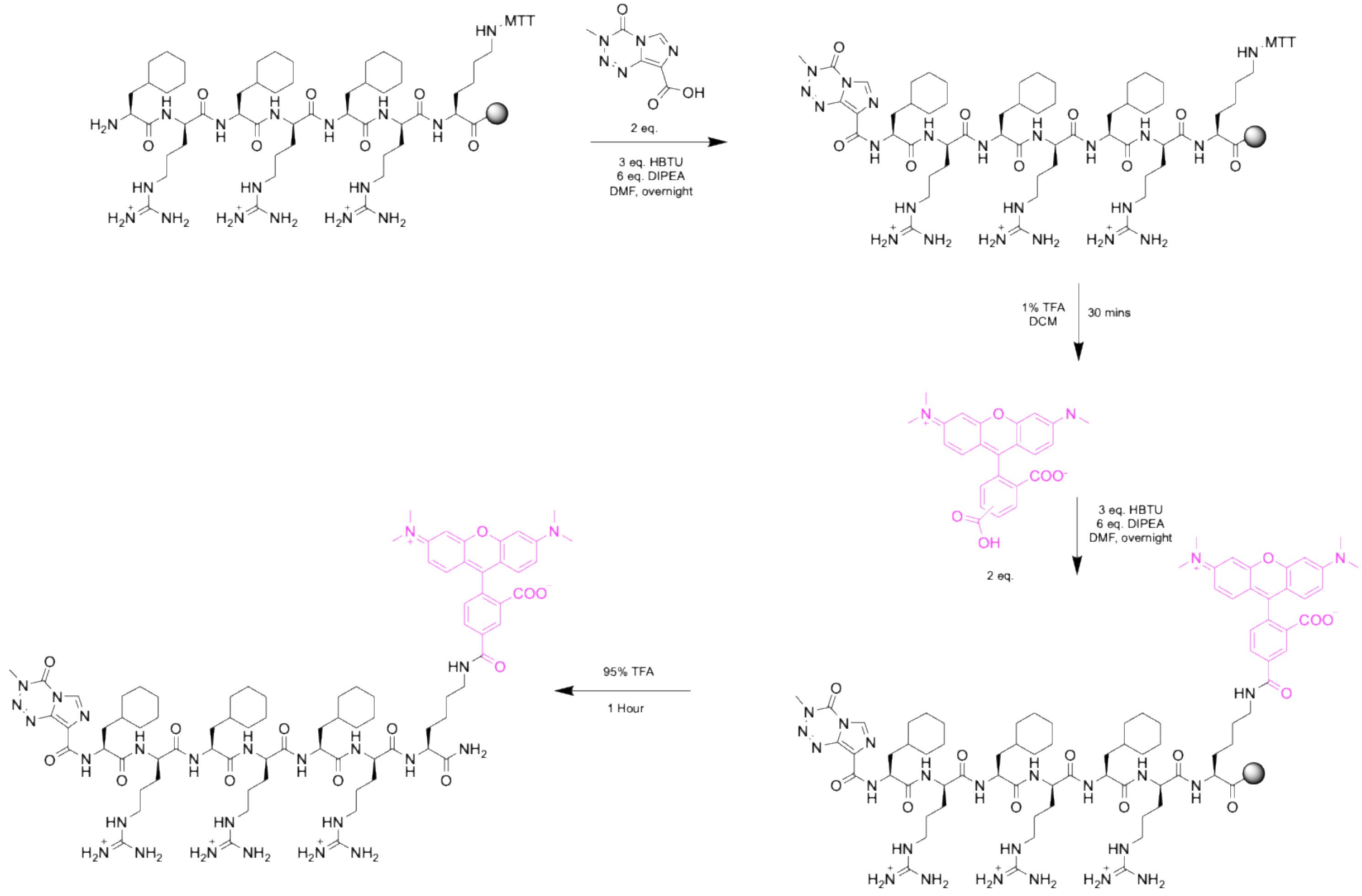

Scheme S2. Synthetic scheme TAMRA-mtTmz probe used for subcellular localization studies using fluorescence microscopy. The sphere represents the solid bead resin containing the peptide until it is cleaved by concentrated TFA after Tmz conjugation. The 5,6-TAMRA isomer was used in the synthesis and is indicated in purple.

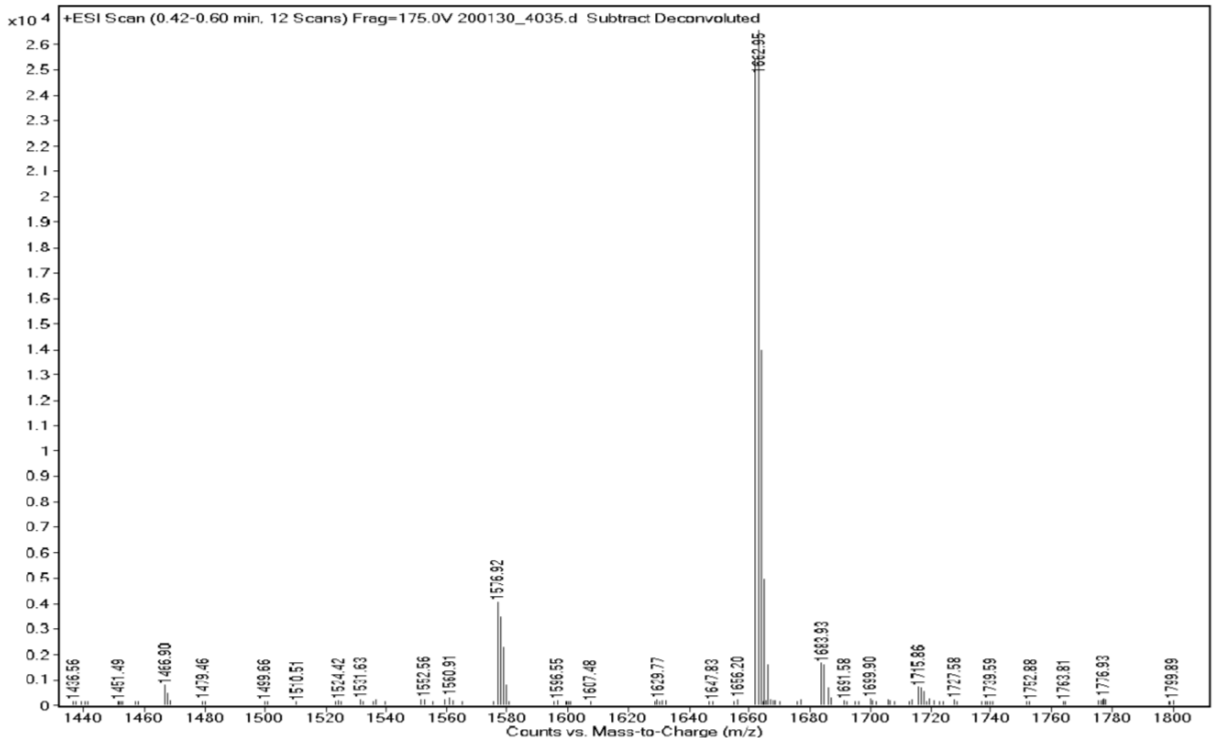

Figure S3. Mass spectrum of TAMRA-mtTmz.

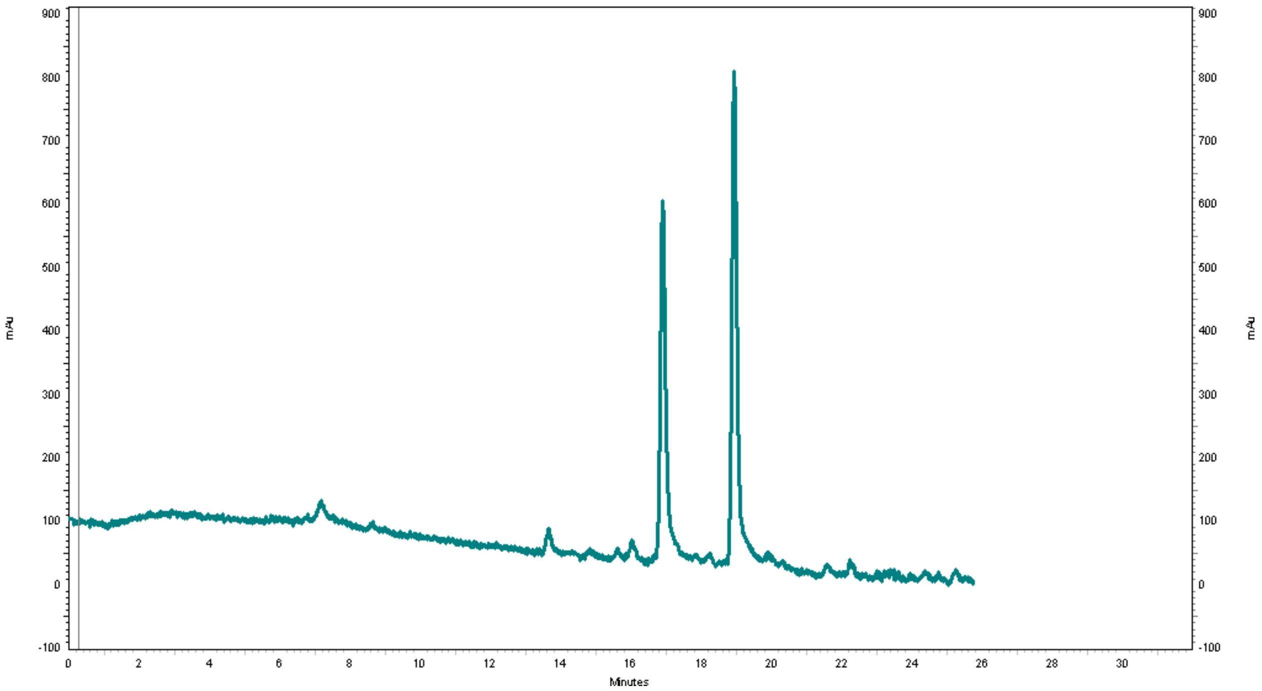

Figure S4. HPLC chromatogram of pure TAMRA-mtTmz. The two peaks correspond to 5,6-TAMRA isomer used for synthesis.

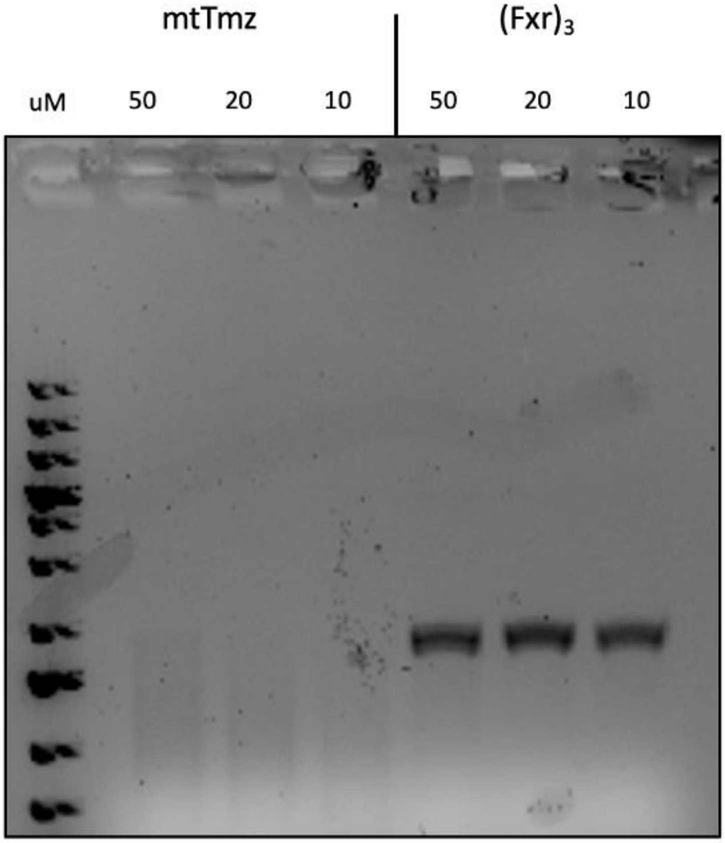

Figure S5. DNA Alkylation assay comparing mtTmz and $(Fxr)_3$.

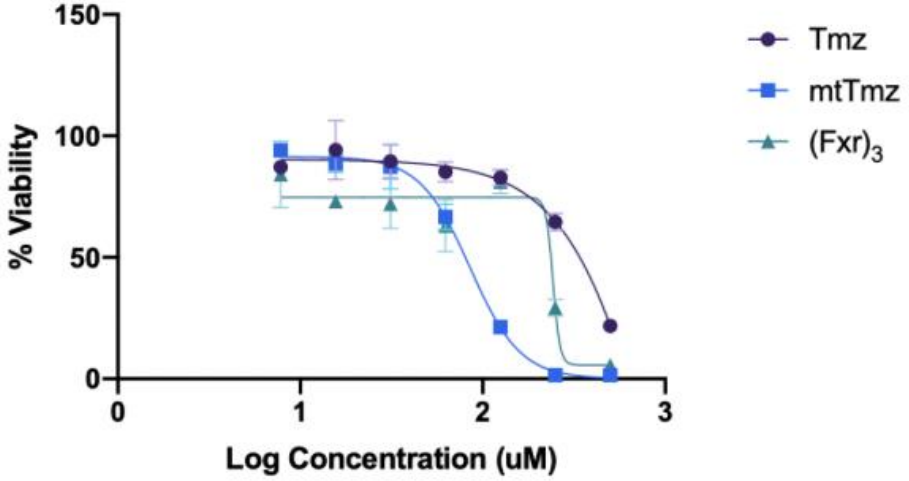

Figure S6. 48-hour cell viability assay in HeLa cells comparing toxicities of Tmz and mtTmz, with $(Fxr)_3$ as a control. Analyzed using CCK8 and normalized to DMSO vehicle control.

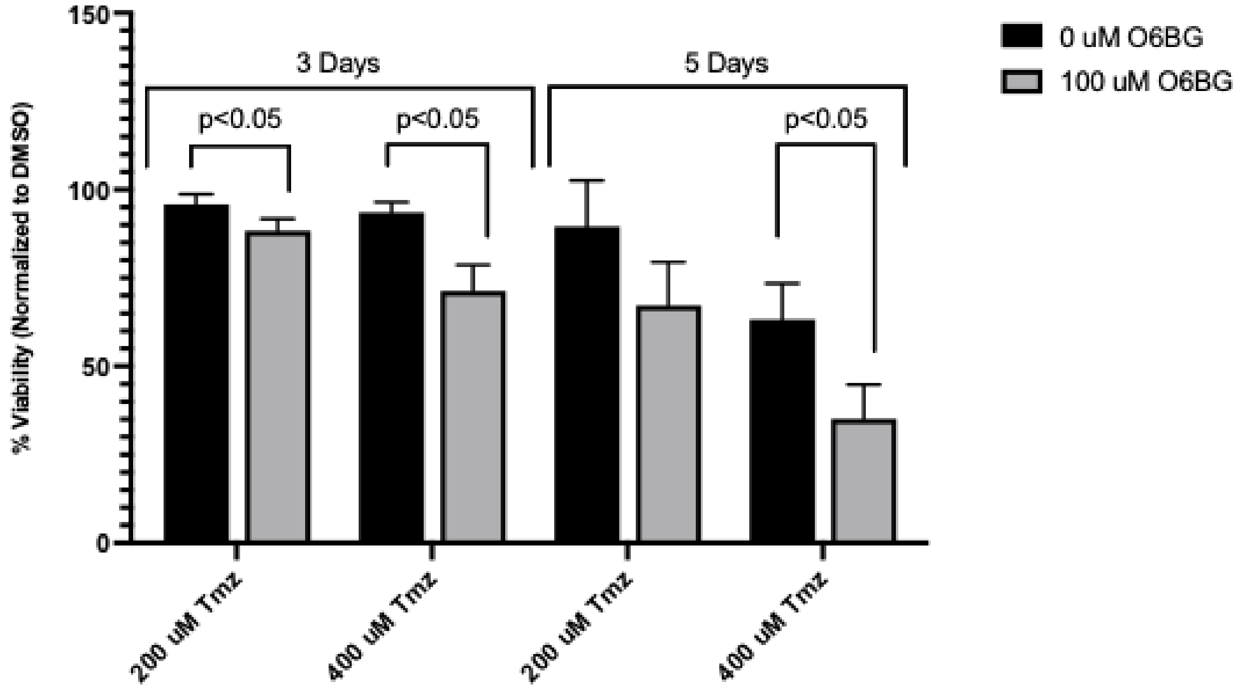

Figure S7. Effect of MGMT inhibitor O^6BG on viability of U251 $MGMT^+$ cells treated with Tmz. 3- and 5-day treatments were performed using 200 and 400 uM Tmz, with and without 3-hour pre-treatment with 100 uM O^6BG.

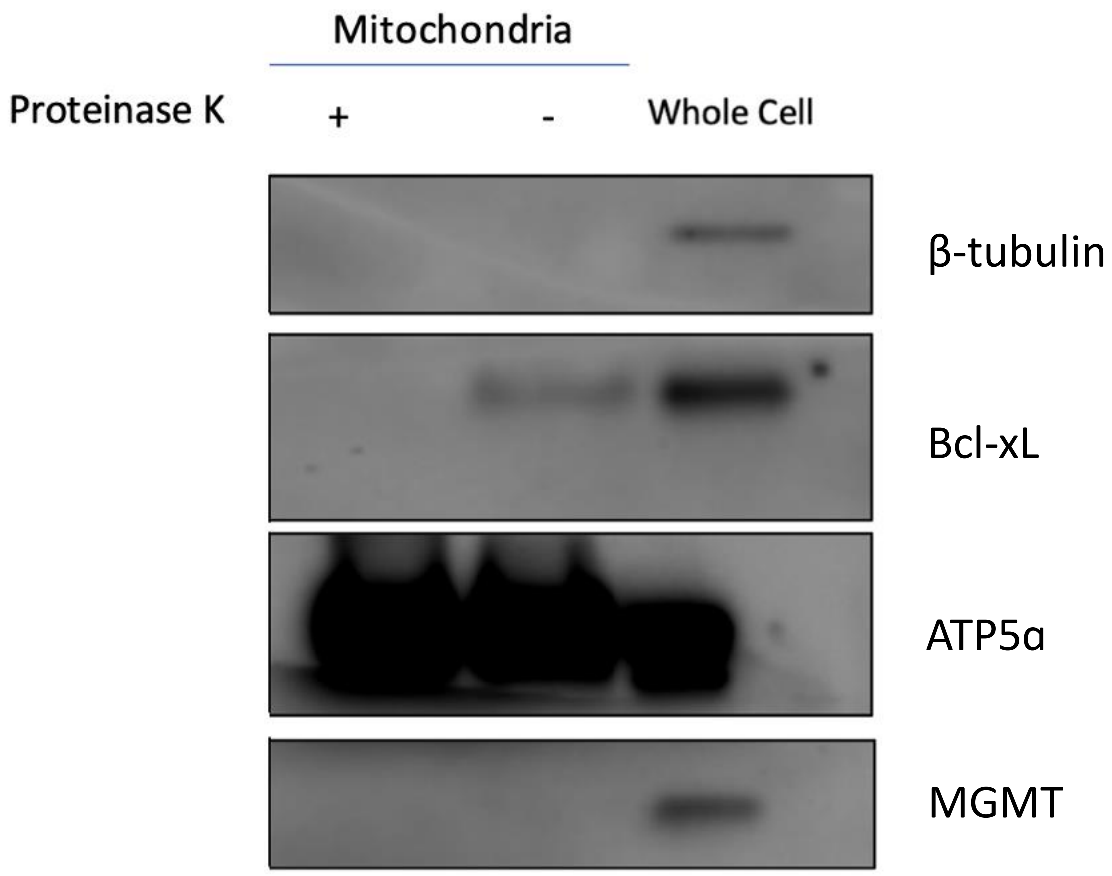

Figure S8. Proteinase K digestion western blot assay. HeLa cells were treated with 50 uM mtTmz for 24 hours, after which mitochondria were isolated and either suspended in buffer alone, buffer containing Proteinase K, or buffer containing both Proteinase K and SDS and separated based on size using SDS-PAGE. Bcl-xL is a mitochondrial outer membrane protein. ATP5α is a mitochondrial matrix protein. β-tubulin acts as whole cell lysate control.
